# Simulation of in vivo dynamics during robot assisted joint movement

**DOI:** 10.1186/1475-925X-13-167

**Published:** 2014-12-16

**Authors:** Evgenij Bobrowitsch, Andrea Lorenz, Nikolaus Wülker, Christian Walter

**Affiliations:** Department of Orthopaedic Surgery, Biomechanics Laboratory, University Hospital Tübingen, 72076 Tübingen, Germany

**Keywords:** Biomechanics

## Abstract

**Background:**

Robots are very useful tools in orthopedic research. They can provide force/torque controlled specimen motion with high repeatability and precision. A method to analyze dissipative energy outcome in an entire joint was developed in our group. In a previous study, a sheep knee was flexed while axial load remained constant during the measurement of dissipated energy. We intend to apply this method for the investigation of osteoarthritis. Additionally, the method should be improved by simulation of in vivo knee dynamics. Thus, a new biomechanical testing tool will be developed for analyzing in vitro joint properties after different treatments.

**Methods:**

Discretization of passive knee flexion was used to construct a complex flexion movement by a robot and simulate altering axial load similar to in vivo sheep knee dynamics described in a previous experimental study.

**Results:**

The robot applied an in vivo like axial force profile with high reproducibility during the corresponding knee flexion (total standard deviation of 0.025 body weight (BW)). A total residual error between the in vivo and simulated axial force was 0.16 BW. Posterior-anterior and medio-lateral forces were detected by the robot as a backlash of joint structures. Their curve forms were similar to curve forms of corresponding in vivo measured forces, but in contrast to the axial force, they showed higher total standard deviation of 0.118 and 0.203 BW and higher total residual error of 0.79 and 0.21 BW for posterior-anterior and medio-lateral forces respectively.

**Conclusions:**

We developed and evaluated an algorithm for the robotic simulation of complex in vivo joint dynamics using a joint specimen. This should be a new biomechanical testing tool for analyzing joint properties after different treatments.

## Background

Robots are very useful tools in orthopedic research. High repeatability and precision of the robotic movement as well as the control of applied forces/torques allow assessment of joint stability after different surgical reconstruction techniques [1], investigation of instability mechanisms of different implants [2] and so on. Additionally, a robot can be applied for measurements of dissipated energy as a frictional characteristic in an entire joint [3].

Osteoarthritis is a common degenerative joint disease, which leads to a loss of the excellent frictional properties of synovial joints such as the knee. The abrasive processes over a lifetime or arthritic inflammatory diseases can lead to cartilage surface degeneration. The result is increased wear and energy dissipation during daily movements [4].

As a result of the rising number of patients suffering from osteoarthritis, there is a need for further development of effective therapeutic approaches. Clinical, histological and imaging tests indirectly described the mechanical improvement of joint cartilage after osteoarthritis treatment. Therefore, tribological tests for mechanical characterization of the cartilage are also required.

Currently, three related tribological methods are prevalent. The first method was the so-called ‘pin-on-disc’ [5], where small cartilage/bone cylinders were cyclically rotated or reciprocated under constant axial load in order to determine the coefficient of friction. The second method was the pendulum. Here, cadaver knees were used as the fulcrum of a pendulum and the amplitude decay was used to calculate the friction coefficient [6, 7]. In the third method, a robot was used to measure friction coefficients in rabbit stifle joints by applying a linear movement [8].

A new method to measure dissipated energy as a friction characteristic in an entire joint was developed in our group [3, 9, 10]. Using a robot, ovine knee joints were physiologically, non-linearly moved under angle and force/torque control [3]. The physiological joint flexion was determined by a passive path. To simplify the measurement method, we used a constant axial load and flexion velocity during the knee flexion [3], but under real conditions, the axial force and also the flexion velocity altered during stance and swing phases in a gait cycle. Taylor et al. described both the flexion motion and contact forces of the ovine knee joint throughout a gait cycle [11].

The aim of this study was to develop and evaluate an algorithm for a robot [3] in order to reproduce the complex in vivo joint dynamics in ovine knee specimens using the data of Taylor et al. [11]. In vitro simulation of in vivo dynamics will provide a new biomechanical testing tool for analyzing joint properties after different treatments.

## Methods

### Robot

A robotic 6-degree-of-freedom setup (KUKA KR 60–3 robot, Augsburg, Germany; reproducibility: ±0.06 mm) including a universal force/torque sensor (ATI UFS: Theta SI1000-120; resolution: 0.25 N and 0.025 Nm) was used to perform axially loaded knee flexion.

### Specimen preparation

**Figure 1:**
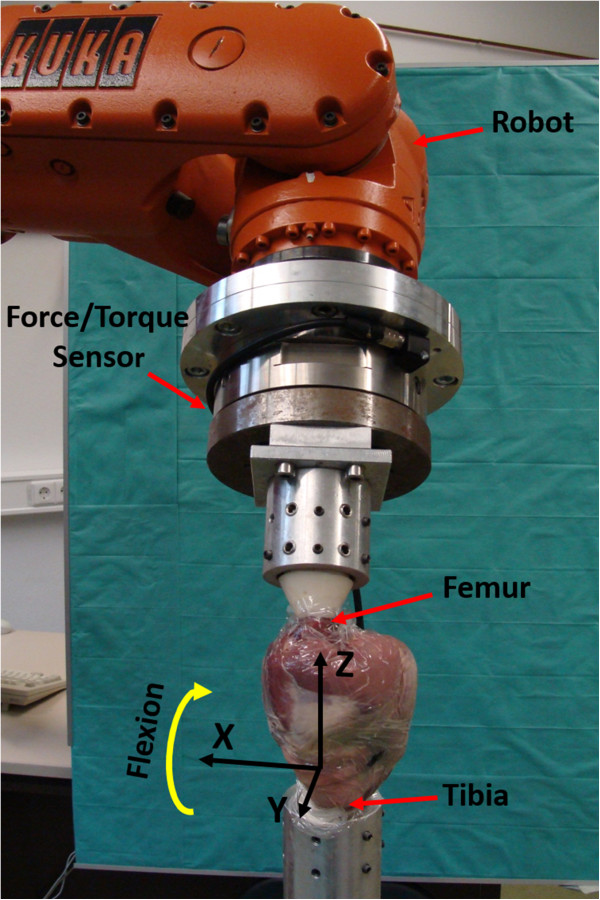
**Experimental setup.** Black arrows represent the tibia coordinate system where X, Y and Z denote medio-lateral, posterior-anterior and axial directions respectively. An ovine knee specimen was flexed by the robot around the medio-lateral X axis.

Four (two left and two right) fresh frozen, healthy, skinless sheep knee joints were obtained post mortem and directly stored at -20°C. Approximate body weights of the four sheep were 30 kg, 30 kg, 30 kg and 35 kg. Prior to testing, the joints were thawed for about 16 hours at room temperature wrapped in a cloth soaked with saline solution. The femur and tibia were resected 20 cm proximal and distal to the joint gap and embedded in a two-component resin (RenCast^©^ FC 53 isocyanate/FC 53 polyol, Gößl & Pfaff GmbH, Karlskron, Germany). The embedded bones were fixed within aluminum cylinders using radial screws around the circumference. For the testing procedure, the tibial cylinder was fixed to a column stand which was connected to the floor while the femoral cylinder was attached to the robotic system. During experiments, the specimens were wrapped in thin polyethylene film to preserve the specimens from drying (Figure 1). After the experiments, the knee joints were dissected to visually control the joint structures.

### Axes definitions

The femoral epicondyles of the knee joint were palpated and marked with a black felt pen. The initial origin of the robotic coordinate systems of the base (tibia, fixed) and tool (femur, moving) were placed in the midpoint between the lateral and medial epicondyle. For the tibial and femoral local coordinate systems, the x-axes were defined from medial to lateral, the y-axes from posterior to anterior and the longitudinal z-axes from distal to proximal. The x-axis for each of the two coordinate systems was a flexion axis specified perpendicular to the respective femoral and tibial longitudinal z-axis, approximately coinciding with the epicondylar axis (Figure 1). Tibiofemoral movement was recorded in the tibia (base) coordinate system.

### Recording of passive knee flexion

Similar to the previous study [3], passive knee flexion was recorded with seven different increments (0.1, 0.2, 0.3, 0.4, 0.5, 0.75 and 1°) for one specimen in order to determine the optimal angular resolution for the knee flexion motion. Passive knee flexion of another three specimens was recorded with the optimal increment. The optimal increment was determined as a minimal increment with which the desired flexion velocity could be performed by the robot.

Passive knee flexion was characterized in a way that it follows the path of minimal resistance. It has been shown that this path of passive flexion was unique for each knee joint and therefore describes the individual unloaded motion of the joint [12, 13]. Passive knee flexion was recorded with the robotic system using a combination of angle controlled and force/torque controlled motion [14]. While the flexion movement around the local femur flexion medio-lateral axis was carried out in angle controlled mode, the remaining five degree of freedom were force/torque controlled at the same time. All forces and torques were specified to be zero (acceptable tolerance for the residual magnitude less than 2 N and 0.2 Nm respectively), except for the axial force (along the tibia longitudinal axis), where 10 N were applied to guarantee a contact of the two joint surfaces [15]. The knee flexion motion was started by approximately 50° of the knee flexion angle which corresponds to the minimal knee flexion angle during the heel-strike recorded by Taylor et al. [11], then the knee was flexed for 30° with the specified increment (until the flexion angle of 80°). Each point of the passive flexion path was recorded after all force/torque conditions were met. Thus, for instance, after recording passive knee flexion with an increment of 0.5°, the path of the passive knee flexion consisted of 61 points (an initial point plus 60 points after rotations with an increment of 0.5°).

In order to optimize the robot movement around the knee flexion axis, the origins of the tibial (base) and femoral (tool) coordinate systems were newly defined in the knee rotation center. Therefore, passive knee flexion was performed twice: the first time using a manually determined knee rotation center and the second time using the calculated knee rotation center. Thereafter, Matlab software (Matlab R2013a, The Math-Works, Inc., Natick, MA, USA) was used for the data processing described below.

The knee rotation center was calculated as a minimal amplitude point [16] during tibiofemoral movement of the first recorded passive knee flexion. Ehrig et al. showed that the minimal amplitude method was less prone to noise [17] than the least-squares algorithm which was used for the same purpose in the previous study [3]. Thus, the robotic flexion axis was corrected to the calculated knee rotation center which corresponded to that optimized axis and the passive knee flexion was recorded again.

### Preparation of dynamics data for a robot-assisted knee flexion simulation

The curves of averaged sheep’s knee flexion and contact axial force were taken from the work of Taylor et al. [11] to simulate an in vivo load on an ovine knee specimen during the flexion. The data of both curves from heel-strike to heel-strike (100% of a complete gait cycle) were corrected at the start and end of the gait cycle in order to avoid a pulse movement of the robot during a juncture of two adjacent gait cycles. To perform this correction, the data of three gait cycles were connected in one curve and processed with a low pass filter and then the corrected data of the second gait cycle were used to interpolate flexion and axial force curves for the robot.

**Figure 2:**
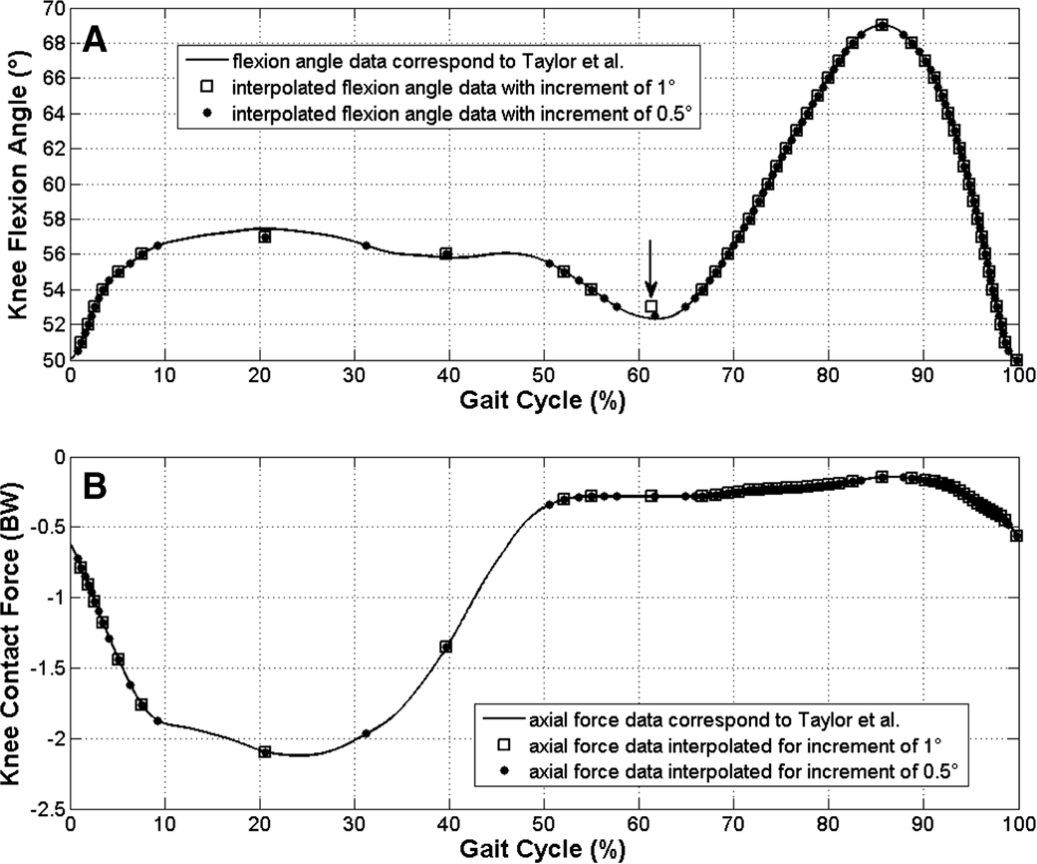
**Data interpolation for the robot.** In order to program the robot movement, knee flexion data were interpolated for the robot (diagram **A**, points and squares) using the flexion data from Taylor et al. [11] (diagram **A**, solid line). If two adjacent, interpolated points at local minima or maxima had the same flexion angle, the midpoint (square in diagram **A** marked with a black arrow) was taken instead of both points (for more information, see the text). Interpolation of axial force data (diagram **B**, points and squares) was performed using the axial force data from Taylor et al. [11] (diagram **B**, solid line) in accordance with the gait cycle data of the interpolated incremental flexion (diagram **A**, points and squares).

The flexion angle data were interpolated for all of the seven increments. An example of the incremental interpolation of the flexion angle data was shown in Figure 2A. If two adjacent, interpolated points at local minima or maxima had the same flexion angle, the midpoint was taken instead of both points (Figure 2A, black arrow) in order to avoid zero angular velocity between the two points.

The absolute angular velocity to rotate the femur around the x-axis by means of the robot (Figure 1) was calculated as a differential of the knee flexion angle data for each of the seven increments. To undergo the differentiation, the end value of the knee flexion angle data was added to the flexion angle data set as its first element in order to avoid shortening of the data set length after the differentiation. Then, the data set of the absolute angular velocity values was normalized to a maximal value from this data set. Thus, to calculate an angular velocity profile for a gait cycle with a desired maximal angular velocity, the set of the normalized angular velocity data had to be multiplied with the desired maximal value.

The axial contact force values in body weight (BW) for each of the seven increments were interpolated from the knee contact force values published by Taylor et al. [11] to corresponding gait cycle values of the flexion angle curves (see the example shown in Figure 2B). Thus, to calculate a set of axial forces for a gait cycle with a desired body weight in Newton (N), the set of the axial contact force data in BW had to be multiplied with the sheep’s body weight in N.

### In vivo loaded knee flexion simulation

The flexion angle, angular velocity and axial force data sets were vectors of the same length. These three vectors of data comprised dynamics information for the simulation of knee flexion over one gait cycle. The loaded knee flexion path of the robot movement consisted of points (spatial positions of the robot) from the previously recorded passive flexion path. In order to reproduce the knee flexion during the gait cycle, we substituted the values of the interpolated flexion angle data for indexes of the corresponding points of the passive flexion path. Thus, the loaded knee flexion path was constructed from points of the passive flexion path to simulate the sheep’s knee flexion.

The robot performed the loaded knee flexion path in a superimposed force torque control mode. Only the axial force was actively simulated (controlled) by the robot. Medio-lateral and posterior-anterior forces resulted as a backlash of knee joint structures. The simulated body weights were 300 N, 300 N, 300 N, and 350 N corresponding to the sheep’s body weights. The in vivo loads were simulated for 200 gait cycles on each of the four knee specimens. The 60 initial gait cycles were considered as pre-conditional cycles, while the last 140 gait cycles were used for further data analysis.

### Experiment with flexion velocity over 10°/s limit

As a precaution of our occupational health and safety department, the angular velocity of the robot was limited to 10°/s. This allowed work with the robot without a protective fence. In order to show the reaction of the robot control to target angular velocities above this 10°/s limit, the knee flexion was performed on one specimen with three different flexion velocity profiles. Three flexion velocity profiles were calculated for maximal flexion velocity of 10, 15 and 20°/s.

### Comparison of simulated forces to in vivo forces

The dynamics data of simulated knee flexion were recorded with a sampling time of 24 ms. In order to calculate the residual error between the simulated force data and the in vivo force data published by Taylor et al. [11], the in vivo force data were interpolated for each percent of the gait cycle while the simulated force data were averaged for a category of 1% during the gait cycle. Thus, the residual error *ϵ* in BW units was calculated as follows:


where m denoted four specimens, n denoted 140 gait cycles. *F*_*ij*_ was the simulated force vector and *F*_*invivo*_ was the in vivo force vector corresponding to Taylor et al. [11]. Both force vectors consisted of 100 elements in body weight units.

The total residual error *ϵ*_*total*_ in BW units was calculated as follows:


where *p* denoted 100 elements of a force vector. *F*_*kij*_ was an element of the simulated force and *F*_*k* _ *invivo*_ was an element of the in vivo force.

### Standard deviation of the simulated forces

The simulated force data were averaged for a category of 1% during the gait cycle. Thus, the gait cycle consisted of 100 elements. For each percent of the gait cycle, a standard deviation of the forces was calculated in body weight units. The total standard deviation *σ*_*total*_ in BW units was calculated as follows:


where , *m* denoted four specimens, *n* denoted 140 gait cycles and *p* denoted 100 elements of a force vector.

## Results

The experiments on all knee specimens were successfully completed. Visual examinations of the specimens before, during and after joint dissection revealed that the bones, ligaments, menisci, joint cartilage and capsule were intact.

### Determination of an optimal flexion increment

**Figure 3:**
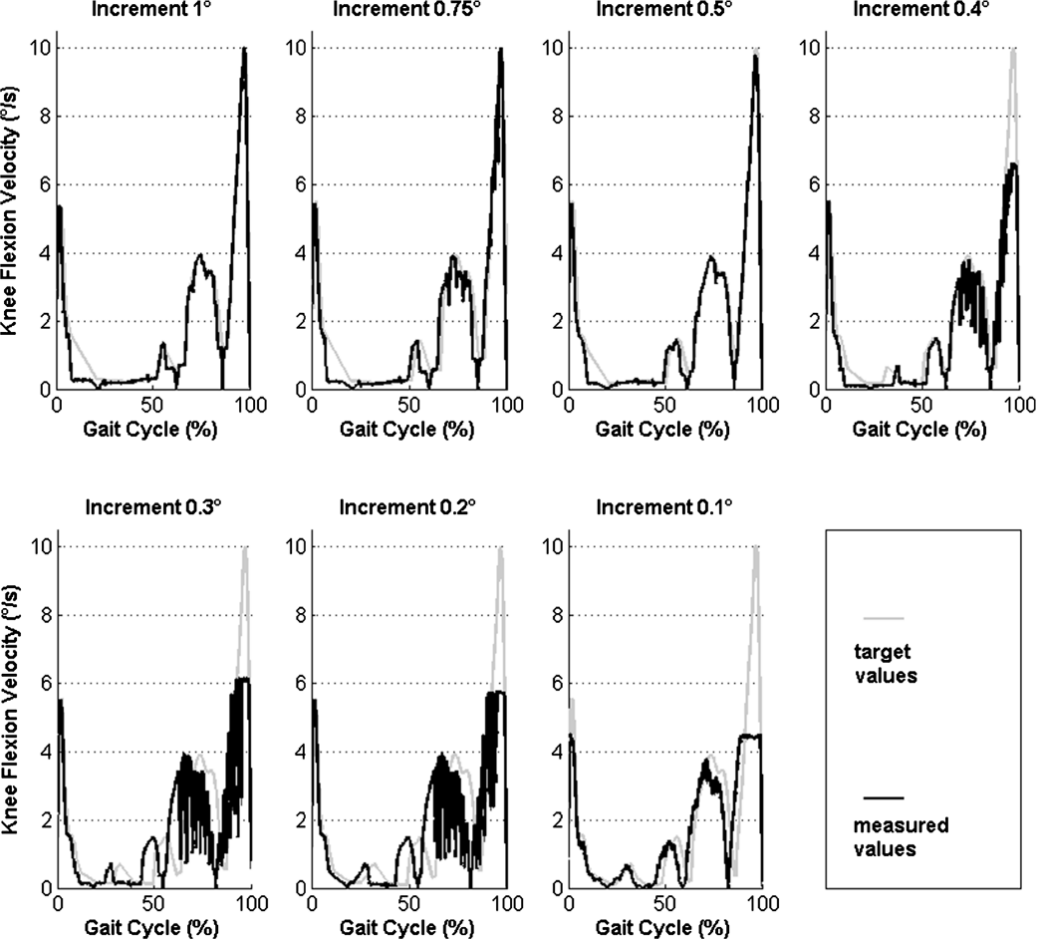
**Determination of an optimal flexion increment.** Knee flexion velocity curves (black) were measured for seven increments. The target velocity profiles were calculated for a maximal value of 10°/s (gray curves). An increment of 0.5° was determined as optimal.

As shown in Figure 3, the maximal velocity of 10°/s was achieved with increments of 1, 0.75 and 0.5°. The measured velocity with smaller increments (0.4, 0.3, 0.2 and 0.1°) showed a reduction in maximal flexion velocity up to 4.5°/s for an increment of 0.1°. Therefore, the increment of 0.5° was defined as the optimal flexion increment.

### Influence of flexion velocities above the 10°/s limit

**Figure 4:**
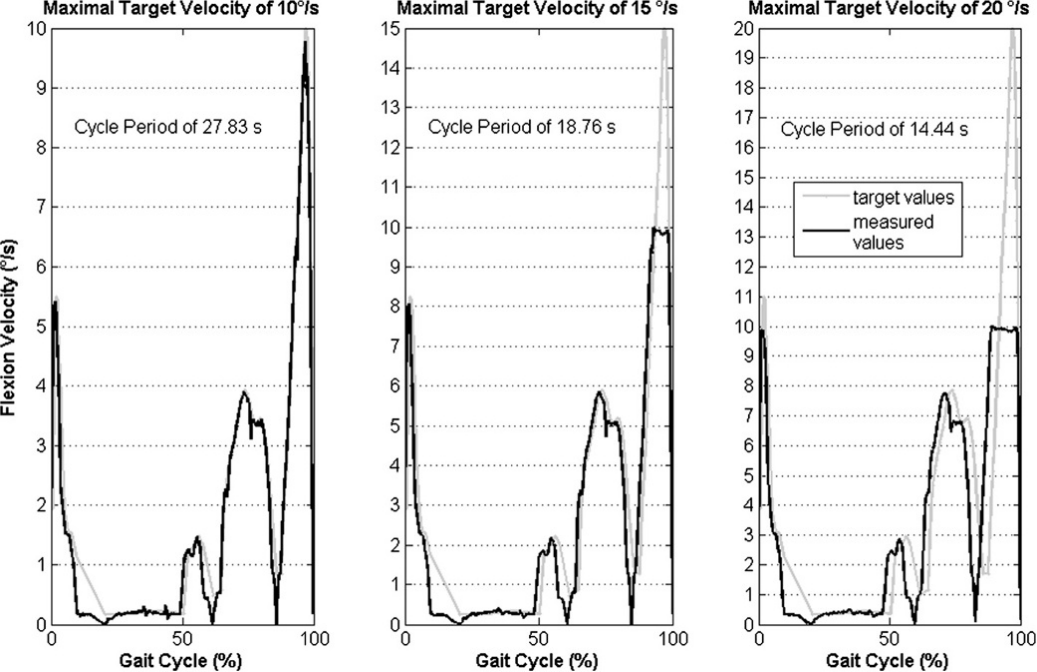
**Flexion velocity profiles above the 10°/s limit.** Knee flexion velocity curves (black) were measured during the simulation of the gait cycle with three different target velocity profiles. The target velocity profiles were calculated for maximal values of 10, 15 and 20°/s (gray curves).

The robot flexed the knee three times with the optimal increment of 0.5°. The first time it flexed with a velocity profile calculated for a maximal velocity of 10°/s, then 15°/s and 20°/s. Therefore, periods of one gait cycle had durations of 27.8, 18.7 and 14.4 seconds respectively. When the predetermined flexion velocity was above the 10°/s limit, the robot flexed the knee at 10°/s (Figure 4 middle and right).

**Figure 5:**
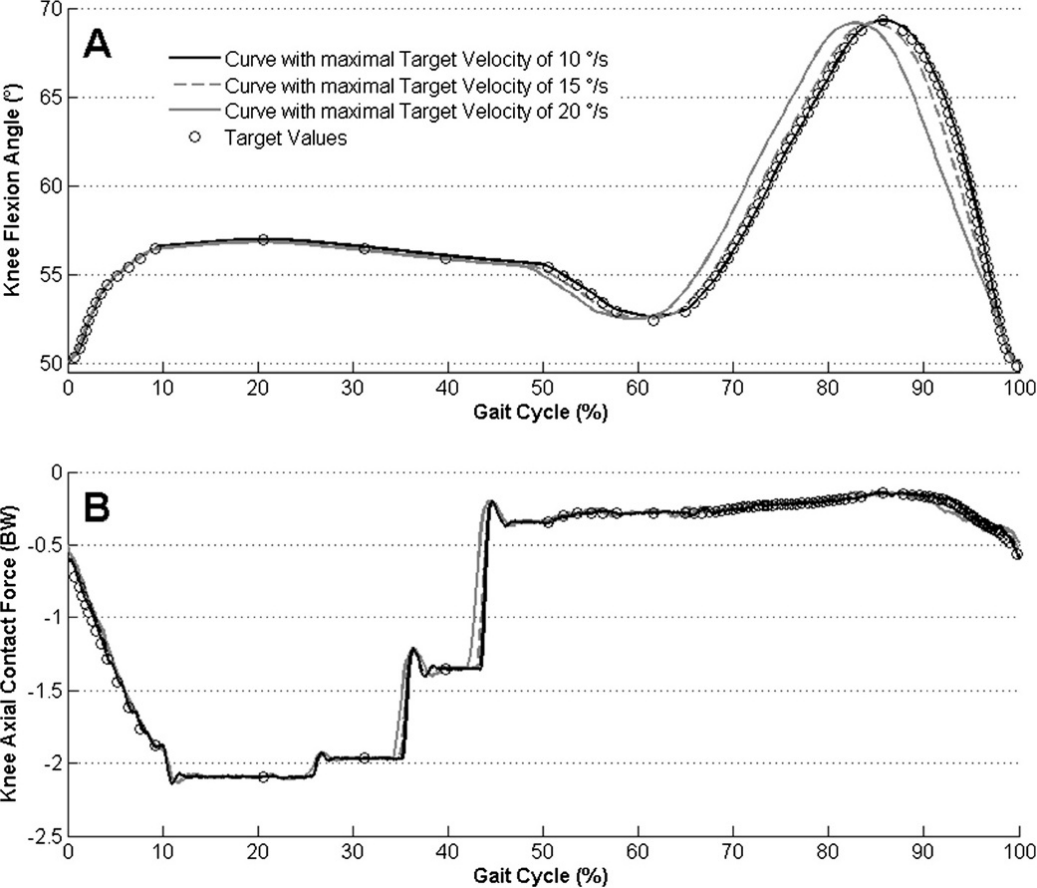
**Influence of flexion velocities above the 10°/s limit. (A)** Knee flexion angle curves and **(B)** axial contact force curves were measured during the simulation of the gait cycle with three different target velocity profiles with maximal target velocity of 10 (black), 15 (gray dashed) and 20 (gray)°/s.

The limitation of the flexion velocity to 10°/s, which had an impact during the swing phase of the gait cycle, caused a left shift of flexion angle curves after 50% of the gait cycle (Figure 5A). In contrast, the axial contact force curves showed a small left shift while all target values were closely passed (Figure 5B).

### Comparison of simulated forces to in vivo forces

**Figure 6:**
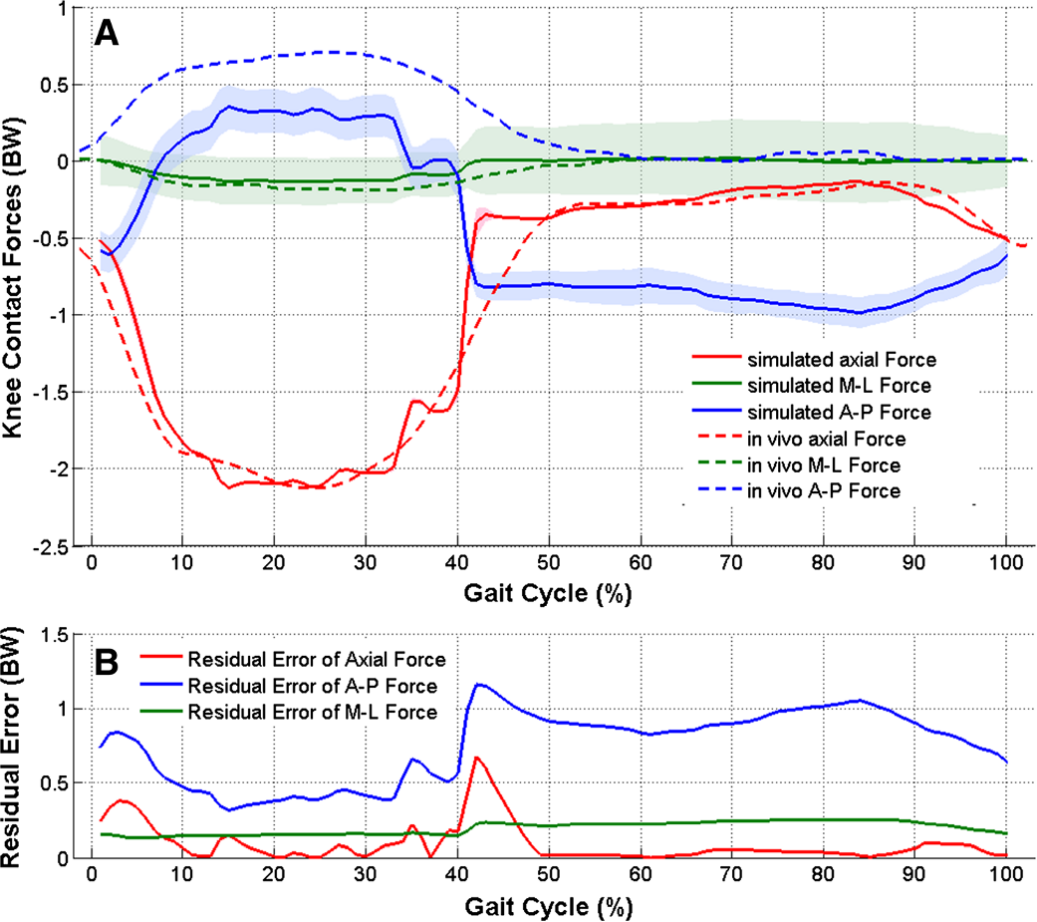
**Comparison of simulated forces to in vivo forces. (A)** Curves of averaged contact forces simulated on four ovine knees (solid lines, the shaded areas along the curves denote ± one standard deviation). The dashed curves represent in vivo forces published by Taylor et al. [11]. **(B)** The residual error curves represent the error between corresponding in vivo and simulated forces.

The total residual error *ϵ*_*total*_ between the in vivo and simulated data resulted in 0.16, 0.21 and 0.79 BW for axial, medio-lateral and posterior-anterior forces respectively. The residual error curves showed that the residual error had increased values at the beginning of the gait cycle and during the end of the stance phase (between 39 and 52%, Figure 6B).

### Standard deviation of the simulated forces

The total standard deviation *σ*_*total*_ resulted in 0.025 BW for the axial contact force. In contrast, the posterior-anterior and medio-lateral forces showed higher total standard deviations *σ*_*total*_ of 0.118 and 0.203 BW respectively.

## Discussion

In our study, we presented an algorithm for the KUKA KR 60–3 robot to simulate the complex flexion movement of an ovine knee including the altering axial load similar to the in vivo dynamics of a sheep’s knee described in the study of Taylor et al. [11]. Knee flexion simulations with different body weights and flexion velocities were performed.

The robot movement was based on points (spatial positions of the robot) recorded during passive knee flexion. Discretization of the flexion path allowed construction of the gait cycle path from points of the passive flexion path. The optimal increment (0.5°) of the flexion movement was determined for the robot. The robot motion control automatically reduced flexion velocity if the points of the flexion path were too close to each other. This effect was observed for increments of 0.4° and smaller (Figure 3).

During the stance phase (from 0 to 54% of the gait cycle [11]), the knee was flexed by about 7°, therefore, the smaller the flexion increment the more points could be used to apply different forces, making the simulation force curve smoother and more detailed. The increment of 0.5° was considered as optimal because the maximally allowed flexion velocity of 10°/s could be achieved. According to Taylor et al. [11], an estimated maximal knee flexion velocity amounted to 270°/s during a 1 m/s walking speed of a sheep. In contrast, our robot flexed the sheep knee approximately 27 fold slower than was measured in vivo [11]. A slow knee joint flexion velocity was well suited to measure changes of the joint-dissipated energy, as was shown during measurements with the knee flexion velocity of 3°/s in the previous study [3].

The use of the velocity profile with maximal target velocity of 20°/s showed the possibility to perform the gait cycle period almost two times faster than the period for the velocity profile with a maximal target velocity of 10°/s (Figure 4). It was done without a strong effect on the axial force simulation (Figure 5B). If the flexion velocity during the swing phase is considered of minor importance, the use of the velocity profile with a maximal target velocity of 20°/s may be appropriate for the faster performance of experiments. This could be important during the simulation of thousands of gait cycles because of specimen autolysis.

There were only two target points (Figure 5) between 39 and 52% of the gait cycle with the biggest axial force difference of almost 1 BW. Therefore, the stepwise force alteration between these points was accompanied by the biggest residual error (Figure 6B). Due to the correction of inconsistencies between the start and end of the in vivo measured knee flexion (see section “Preparation of dynamics data for a robot-assisted knee flexion simulation”), the simulated flexion and therefore the simulated axial force could differ from the in vivo curves of Taylor et al. [11]. This could explain the increased residual error at the beginning of the gait cycle (Figure 6B).

Only the axial force was actively simulated by the robot. The medio-lateral and posterior-anterior forces resulted as a backlash of knee joint structures. The highest residual error was observed for the posterior-anterior force (*ϵ*_*total*_ = 0.79 BW). We assume that in the absence of active muscles, the patellar or hamstring tendons could not contribute to the posterior-anterior force. Therefore, in our experimental set-up, the measured posterior-anterior force differed from in vivo values (Figure 6A). For future applications of our robot system, it could be very interesting to analyze the contribution of passive joint structures like ligaments, capsule, etc. to the development of posterior-anterior and medio-lateral forces, which are very important for joint stability. The Orthoload database (http://www.orthoload.com) can be used to transfer in vivo measured forces to our robot system. Then, with our robot system, it could be possible to analyze, for instance, soft tissue balancing after total joint replacement.

The sheep model is a well-established model for analyzing biological responses during healing [18, 19]. In the future, we intend to use the robot system presented for the in vitro establishment of an enzymatically induced osteoarthritis model. Hollande et al. found a significant reduction of collagen II content in cartilage from osteoarthritis knees in comparison to healthy knees. They explained this phenomenon through increased hydration which took place more on the cartilage surface than in the deep zone of the cartilage [20]. We will simulate the in vivo knee dynamics in order to reveal the relationship between the number of gait cycles and joint cartilage damage (dissipated energy outcome) after injection of a certain quantity of enzyme. Thus, we will study the possibility of transferring the in vitro osteoarthritis model to an in vivo healing model of osteoarthritis.

## Conclusion

The study presented an algorithm to simulate in vivo dynamics on ovine knees by the KR 60–3 robot. We used discrete points of the passive flexion path to construct a complex knee flexion motion similar to the motion measured on sheep in vivo. The robot applied an in vivo-like axial force profile with high reproducibility during the corresponding knee flexion. Posterior-anterior and medio-lateral forces were detected by the robot as a backlash of joint structures. Their curve forms were similar to the curve forms of corresponding in vivo measured forces, but in contrast to the axial force, they showed higher standard deviation and total residual error.
